# Growth, profitability, nutritional, and anti-nutritional properties of seven *Manihot esculenta* Crantz (cassava) varieties as affected by arbuscular mycorrhizal fungi

**DOI:** 10.1016/j.heliyon.2024.e36371

**Published:** 2024-08-22

**Authors:** Mbassi Manga Gilbert Ghislain, Djontzo Toche Emmilienne, Essono Damien Mari, Adamou Souleymanou, Fokom Raymond, Nouhou Abassi, Noah Guy, Sonkeng Aurelie, Nwaga Dieudonné, Fokou Elie

**Affiliations:** a Institute of Fisheries and Aquatic Sciences, University of Douala, Cameroon; b Soil Microbiology Laboratory, the Biotechnology Centre, University of Yaoundé I, Cameroon; c Laboratory of Food Science and Metabolism, University of Yaoundé I, Cameroon; d Faculty of Agronomy and Agricultural Sciences, University of Dschang, Cameroon

**Keywords:** Cassava varieties, Yield, Profitability, AM fungi, Cyanides

## Abstract

Despite a range of methods used to promote modern agriculture with several outcomes, food quality and availability problems remain. This work aims to evaluate the effect of AM fungi inoculation on the growth, yield, nutritional, and antinutritional properties of 7 varieties of cassava. Growth characteristics, yields, rentability, nutritional, and antinutritional of tubers of each treatment were determined at harvest. All the cassava varieties used form a symbiosis with AM fungi at various frequencies, with the I090590 variety being the best (61.66 %). The best amount of chlorophyll, carotenoid, and height of plants were recorded at 9 months old. The 96/1414, TME/693 and MD varieties respectively show the best amount of chlorophyll, size, and carotenoids at 9 months old. Following AM fungi inoculation, an increase in the content of chlorophyll, size, and carotenoids was recorded for all the varieties with the best rate attributed respectively to 92/0326, MD, and 92/0326. Tuber yields vary significantly depending on the cassava varieties, with the best (56.16 t/ha) recorded for the I090590 variety. Following inoculation with AM fungi, a significant increase in yields was recorded, with the best ratio (2.7) obtained with the AE variety. The I090590 variety shows the best yield and by then the most profitable. Inoculation with AM fungi leads to a significant increase in the sugar, protein, fibre, and phosphorus content of all cassava varieties, with the best ratios obtained in 96/1414, 01/1797, and I090590 varieties respectively. Similarly, the inoculation of cassava varieties with AM fungi leads to a significant reduction in the content of cyanides, oxalates, and phytates. The best ratio of reduction for cyanide was 1.91 for the MD variety. AM fungi inoculation is an important way to ensure safe, exponential production and high economic profitability of foodstuffs.

## 1 Introduction

Cassava (*Manihot esculenta* Crantz) is a perennial shrub from South America origin, that was introduced to tropical regions of Asia and Africa [[1], [2], [3]]. It is a carbohydrate-rich food source able to feed up to 800 million people in the world and is a raw material for starch, bioethanol, and several bio-based products industries [4,5]. Breeding practice, one of the current focuses of scientists to improve plant diversity and production also involves cassava with an important improvement of outcomes food availability highlighted by the current situation of demographic explosion [6]. On the other hand, fertilisation with its diversity contributes to boosting plant production including cassava. Although this approach shows promising results regarding yields and resistance to various pests in plants, it raises the problem of quality of food quality with consequences on human health, resistance to many pests initially under control, and complicates the production system of plants [7,8]. Additional strategies directed improving plant growth and the insurance of a safe environment are actual. This includes safe fertilization fertilisation, appropriate phytochemicals-soil surrounding plant roots, and more. Improving productivity through novel cropping system approaches that integrate in-situ soil bio-fertility improvement techniques is bit by bit gaining priority. Soil microbes that improve nutrient availability and uptake efficiency like AM fungi are therefore valuable alternatives directed for the purpose. Plant adaptations to various ecological systems worldwide are always subject to factors, mainly their ability to develop mutualistic interactions with symbionts [9,10]. Arbuscular mycorrhiza is the oldest widespread association between AM fungi and plant roots, with exchange fluxes from plant part to AM fungi being photosynthates, while fungi part supply plants with essential minerals, including phosphorus and nitrogen [9,[11], [12], [13]]. Additionally, this symbiosis was shown to improve plant growth, production, and tolerance to various biotic and abiotic stresses [[14], [15], [16]]. A growing interest in AM fungi is now the focus of several research programs, and are advised as bio-fertilizers to enhance crop quality and productivity under the ever-changing climate conditions, the biotic and abiotic difficulties to ensure sustainable agriculture [17,18]. Cassava is a highly mycotrophic plant with high benefits to the plant [19,20]. Inoculation of cassava plants with AM fungi positively impacts its growth physiology and yield, with a notable increase in compounds being carotenoids, sugar, proteins, and volatile compounds content [[20], [21], [22]]. The objective of this study was to assess the effect of AM fungi inoculation on the growth, nutritional, anti-nutritional properties, yield, and profitability of seven cassava varieties grown in field conditions.

## 2 Materials and methods

### 2.1 Plant materia and biofertilizer

The seed of cassava varieties used in this work was provided by the International Institute of Tropical Agriculture (IITA), and ′the National Institute of Agricultural Research for Development (NIAR) in Cameroon. They include. Akoa Essame (AE), Mbong Doux (MD) (wild varieties), and I09059, (outcome of breeding) provided by NIARD; 96/1414, 92/0326, 01/1797, and TNE/693 (outcome of breeding) provided by IITA. Characteristics of the seeds are presented in Table 1.

**Table 1:** Characteristics of cassava varieties used in this study. AE, MD, I09059, 96/1414, 92/0326, 01/1797, TNE/693 are cassava varieties.

Varieties	Cycles (month)	Origins	Yields (t/ha)	Inner tubers colors	Steam colors
AE	12	NIARD	30–40	White	Bright red
MD	12	NIARD	30–40	Yellow	Milk-white
I090590	12	NIARD	30–40	Yellow	Reddish
96/1414	12	IITA	30–40	White	Reddish
92/0326	12	IITA	30–40	White	Reddish
January 1797	12	IITA	30–40	White	Bright-red
TME/693	12	IITA	30–40	Yellow	Milk-white

IITA= International Institute of Tropical Agriculture, NIARD= National Institute of Agricultural Research for Development. T/ha = tonnes/hectare.

The mycorrhizal inoculum was provided by the Laboratory of Soil Microbiology of the Biotechnology Centre of the University of Yaoundé 1 in Cameroon. It is the composition of 04 a.m. fungi species isolated from local soils and tested following [23].

### 2.2 Study area and experimentation

The field experiment was conducted at the experimental area of the Yaoundé I University campus in Cameroon. The experiment has 11°31′00″ E of longitude and 3°52′00″ N of latitude. The site is located between 500 and 900 m height from the sea level within the 5th agro ecological zone of Cameroon, characterized by a bimodal rainfall pattern with mean precipitation of 1500 mm and temperature of 28 °C. The on-site soil is a Sandy-clay-loam complex, with physiochemical characteristics including; Organic matter (3.62 %), C/N ratio (20.5), cation exchange capacity (29 meq/100 g) and pH of 5.2. After clearing, the treatment was arrange following a completely randomized block design. There are 14 treatments including 7 control treatments and 7 a.m. fungi inoculated. There are three replications for each treatment. at all, 14*3 individual repetitions are recorded. The experiment cover 4*2 m for each replication. interspace between subplots as well as blocks was 1m. On each subplot, 12 pockets of 20 cm were deep for planting purposes. Sticks of each cassava variety were cut into pieces of 20 cm ready for planting. Chronologically, the control treatment without AM fungi was first planted with 200g of sterilized inoculum per pocket. For AM fungi treatments, the pocket of each plot received 200 g of mycorrhizal inoculums containing 500 spores per pocket. All plots were naturally watered during the experiment.

### 2.3 Growth characteristics of cassava plants

#### 2.3.1 Evaluation of root colonization, chlorophyll, carotenoid content, and plant height

Fresh differentiated leaves from 3-, 6- and 9-month-old cassava plants were collected at the third and fourth leaves and analyzed for total chlorophyll and carotenoid concentration. One gram of circular disks of 5 mm diameter, were punched from the leaf portion and grinding in a mortar with 10 ml of 90 % ethanol, 2 g of pure Silica Quartz, and 0.5 g of calcium carbonate. The extract was filtered using a glass funnel and collected in a conical flask. Quantitative measurements for chlorophyll and carotenoids were carried out using a spectrophotometer (Jenway 6405 UV/vis). absorbencies were recorded at 662, 644, and 445 nm, respectively.

Fresh roots collected at the rhizosphere of two-month-old plants of each treatment were cut into small pieces, mixed with 10 % KOH, and boiled for 30 min in a water bath. They were then rinsed with tap water and acidified with 5 % HCl at room temperature. Finally, the roots were stained by a solution containing 875 ml lactic acid, 63 ml glycerine, 63 ml tap water (14-1-1), and 0.1 g acid fuchsine for 12 h at room temperature and then de-stained with 5 % lactic acid. Stain roots of each treatment were observed at 100–400 magnification under a Nikon YS100 microscope. Structures characteristic of AM fungi infection were recorded for each root fragment and the number of infections was expressed as a percentage [24].

Plant size measurement was done with a graduated tape in cm and record. Three randomly chosen plants at 3, 6, and 9 months old of each replication per treatment were subject to the operation. The support was placed at the base of the plant and its height was recorded at the canopy.

#### 2.3.2 Cassava yield, and profitability

Twelve months after sowing, tubers were harvested following repetitions, and treatments. Tubers’ weights were measured using a scale and record. The yield per treatment was calculated in t/ha following [25].

$$Yield\;\left(t/ha\right)=\left(\frac{mean\;weight\;per\;treatment\;\left(t\right)}{\mathrm{mean}\;\mathrm{plot}\;\mathrm{area}\;\mathrm{per}\;\mathrm{treatment}\;\left(\mathrm{ha}\right)}\right)$$

The average cost of a tone of cassava tuber in the local market is 200,000 XAF. The profitability of potential cassava producers was calculated using the following formula.

Profitability(XAF)=(200000)*Yield

t = tones, ha = hectare, XAF = local Currency.

#### 2.3.3 Proximate analysis

The ash content was determined after calcination of tuber flours of each treatment following [26]. The lipid content of the tuber floor was evaluated by the weight difference of samples after extraction in hexane solvent [27]. Crude protein (nitrogen x 6.25) was determined after mineralization in hot sulphuric acid, and nitrogen titration following [28]. Total sugar content was determined after extraction with ethanol 95 % with a spectrophotometer following [26]. Phosphorus content was determined with a spectrophotometer after digestion with hot concentrated sulphuric acid following [29]. Crude fiber contents were determined after extraction and calcination following [26].

#### 2.3.4 Antinutrient analysis

The oxalate content of flour from cassava tubers was determined by titration with KMnO_4_ after digestion of the sample in a water bath with 3M sulphuric acid for 1 h following [30]. The phytate content of flour from cassava tubers was determined by titration with iron III solutions after the digestion of the sample with 2 % acid chlorite for 3 h [31]. Saponin content of cassava tubers was determined by weight difference after extraction [31]. The cyanide content of cassava tubers was determined according to the protocol described in Ref. [32].

### 2.4 Statistical analysis

Data were subjected to analysis of variance (ANOVA) procedures. Means were separated between treatments with the Fisher's Least Significant Difference (LSD) test at 5 % level of probability, using a Stat-graphic Plus, version 5.0 (SIGMA PLUS) computer package. Comparisons were made between treatments and variety. The Statistical Package for Social Sciences (SPSS) program was used to assess correlations between recorded data.

## 3 Results

**Fig. 1:**
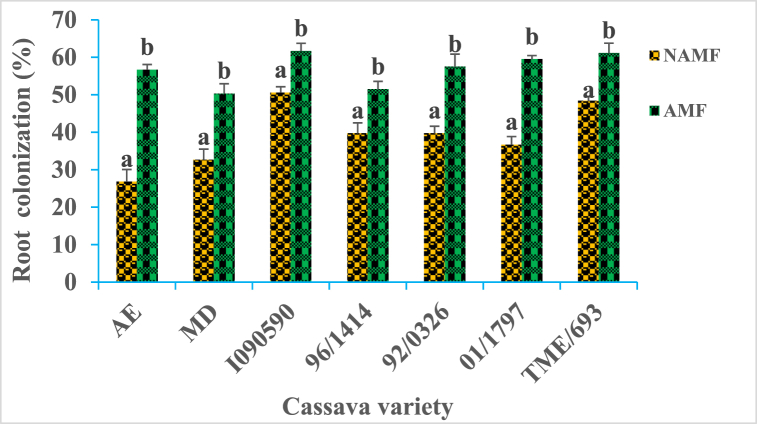
Capacity of AM fungi to form symbiosis with seven cassava varieties in field conditions. *AMF* = *arbuscular mycorrhizal fungi, NAMF* = *none arbuscular mycorrhizal fungi. AE, MD, I090590, 96/1414, 92/0326, 01/1797, and TME/693 are cassava varieties. Bars for each variety with the same letter are not significantly different at P < 0.05.*

Structures characteristic of AM fungi symbiosis were observed in the roots of cassava plants of both inoculated and non-inoculated. However, the percentage of root colonization was significantly (P ≤ 0.05) increased with respect to the variety in inoculated plants compared to none inoculated (Fig. 1). The increase ratios of root colonization vary from 1.21 to 2.03 respectively for 96*/*1414, and AE varieties.

The total leaf chlorophyll content was shown to significantly increase with age for all the varieties of cassava. A significant difference (P ≤ 0.05) with respect to the inoculation status of the plant was recorded for all the varieties of cassava plants (Table 2). The best ratios of chlorophyll content increases were observed at 9 months old, with ratios ranging from 1.25 to 2.28 values respectively for the MD and 92/0326 varieties. At that period of growth, the first three increase ratios were 2.28, 2.03, and 1.95 respectively for 92/0326, 01/1797, and 96/1414 varieties respectively.

**Table 2:** Leaf chlorophyll content variation of cassava varieties with age and AM fungi inoculation in field conditions.

Chlorophyll (mg/g)			
Varieties	3 months	6 months	9 months
AE (AMF)	34.09 ± 0.18^b^	66.84 ± 0.08^b^	106.76 ± 0.19^b^
AE (NAMF)	23.96 ± 0.12^a^	37.32 ± 0.04^a^	77.77 ± 0.05^b^
MD (AMF)	23.50 ± 0.08^b^	48.49 ± 0.46^b^	84.70 ± 0.08^b^
MD (NAMF)	21.09 ± 0.19^a^	41.51 ± 0.04^a^	67.32 ± 0.12^a^
I090590 (AMF)	18.64 ± 0.52^b^	58.07 ± 0.26^b^	94.67 ± 0.06^b^
I090590 (NAMF)	13.17 ± 0.08^a^	34.01 ± 0.00^a^	57.69 ± 0.05^a^
96/1414 (AMF)	26.78 ± 0.46^b^	41.32 ± 0.46^b^	156.25 ± 0.08^b^
96/1414 (NAMF)	20.77 ± 0.29^a^	20.47 ± 0.42^a^	79.86 ± 0.05^a^
92/0326 (AMF)	34.49 ± 0.12^b^	69.52 ± 0.04^b^	103.71 ± 0.08^b^
92/0326 (NAMF)	25.21 ± 0.07^a^	39.87 ± 0.09^a^	45.39 ± 0.08^a^
January 1797 (AMF)	28.96 ± 0.06^b^	54.71 ± 0.08^b^	126.22 ± 0.05^b^
January 1797 (NAMF)	16.54 ± 0.12^a^	29.52 ± 0.16^a^	62.03 ± 0.17^a^
TME/693 (AMF)	28.83 ± 0.18^b^	84.59 ± 0.06^b^	120.46 ± 1.25^b^
TME/693 (NAMF)	17.95 ± 0.03^a^	51.61 ± 0.68^a^	68.63 ± 0.23^a^

Data in Column for each variety at the same period followed by the same letter are not significantly different at P < 0.05. level. (NAMF) = Control; (AMF) = Mycorrhizal. Cassava varieties: Akoa Essama (AE); Mbong doux (MD); I090590; 96/1414; 92/0326; January 1797; TME/693.

The heights of the cassava plant were shown to significantly increase with age for all the varieties. A significant difference (P ≤ 0.05) with respect to the inoculation status of the plant was recorded for all the varieties of cassava plants within the growth (Table 3). The best ratios of height increases were observed at 6 months old, with ratios ranging from 1.02 to 1.41 values respectively for the January 1797 and MD varieties. At that period of growth, the first three increase ratios were 1.41, 1.39, and 1.34 respectively for MD, AE, and 92/0326 varieties respectively.

**Table 3:** Influence of age, AM fungi inoculation on stem height of cassava varieties in field conditions.

Heights (cm)			
Varieties	3 months	6 months	9 months
AE (AMF)	35.5 ± 1.32^b^	140.33 ± 1.53^b^	227.33 ± 2.52^b^
AE (NAMF)	19.67 ± 1.53^a^	100.67 ± 3.05^a^	193.33 ± 2.89^a^
MD (AMF)	75± 2^b^	250.67± 2^b^	304.67 ± 4.51^b^
MD (NAMF)	60± 2^a^	178± 1^a^	272.67 ± 2.52^a^
I090590 (AMF)	67.67 ± 4.04^b^	154± 1^b^	245.33 ± 2.52^b^
I090590 (NAMF)	37.67 ± 8.62^a^	144 ± 2.65^a^	232.67 ± 2.52^a^
96/1414 (AMF)	38.67 ± 2.42^b^	142.67 ± 2.52^b^	214.67 ± 4.22^a^
96/1414 (NAMF)	23.67 ± 2.52^a^	115.67 ± 1.53^a^	210.67 ± 7.04^a^
92/0326 (AMF)	41.17 ± 8.48^b^	120.33 ± 1.53^b^	198.33 ± 1.53^b^
92/0326 (NAMF)	31.67 ± 7.64^a^	88.67 ± 2.08^a^	164.67 ± 1.53^a^
January 1797 (AMF)	78.33 ± 6.51^b^	177.67 ± 5.13^a^	245.67 ± 2.52^b^
January 1797 (NAMF)	58.33 ± 7.62^a^	174.33 ± 1.16^a^	227.00 ± 2.00^a^
TME/693 (AMF)	77 ± 5.29^b^	233.67 ± 0.57^b^	318.67 ± 2.08^b^
TME/693 (NAMF)	59 ± 3.61^a^	200.33 ± 1.53^a^	308.00 ± 1.00^a^

Data in Column for each variety at the same period followed by the same letter are not significantly different at P < 0.05. level. (NAMF) = Control; (AMF) = Mycorrhizal. Cassava varieties: Akoa Essama (AE); Mbong doux (MD); I090590; 96/1414; 92/0326; January 1797; TME/693.

The carotenoid content was shown to significantly increase with age for all the varieties of cassava. A significant difference (P ≤ 0.05) with respect to the inoculation status of the plant was recorded for all the varieties of cassava plants (Table 4). The best ratios of carotenoid content increases were observed at 9 months old. Increase ratios range from 1.09 to 2.67 values respectively for the MD and 92/0326 varieties. At that period of growth, the first three increase ratios were 2.67, 1.86, and 1.73 respectively for 92/0326, TME/693, and I090590 varieties respectively.

**Table 4:** Tuber carotenoid content of cassava varieties in field conditions following AM fungi inoculation and plant age.

Carotenoids (mg/g)			
Varieties	3 months	6 months	9 months
AE (AMF)	10.60 ± 0.09^b^	20.94 ± 0.05^b^	48.54 ± 0.10^b^
AE (NAMF)	7.54 ± 0.05^a^	12.95 ± 0.01^a^	29.92 ± 0.06^a^
MD (AMF)	8.84 ± 0.09^b^	26.88 ± 0.08^b^	41.64 ± 0.03^b^
MD (NAMF)	7.49 ± 0.03^a^	19.55 ± 0.17^a^	38.05 ± 0.047^a^
I090590 (AMF)	15.32 ± 0.05^b^	19.34 ± 0.24^b^	55.01 ± 0.66^b^
I090590 (NAMF)	3.74 ± 0.05^a^	17.96 ± 0.04^a^	31.8 ± 0.05^a^
96/1414 (AMF)	6.14 ± 0.05^b^	21.94 ± 0.16^b^	44.35 ± 0.03^b^
96/1414 (NAMF)	4.57 ± 0.12^a^	10.90 ± 0.04^a^	36.89 ± 0.02^a^
92/0326 (AMF)	11.87 ± 0.23^b^	15.25 ± 0.05^b^	54.46 ± 0.07^b^
92/0326 (NAMF)	6.61 ± 0.07^b^	11.14 ± 0.12^a^	20.39 ± 0.04^a^
January 1797 (AMF)	7.88 ± 0.07^b^	31.67 ± 0.05^b^	54.30 ± 0.95^b^
January 1797 (NAMF)	4.84 ± 0.11^b^	12.90 ± 0.09^a^	31.08 ± 0.02^a^
TME/693 (AMF)	8.63 ± 0.09^b^	40.03 ± 0.02^b^	51.09 ± 0.05^b^
TME/693 (NAMF)	6.52 ± 0.09^a^	30.69 ± 0.19^a^	27.24 ± 1.09^a^

Data in Column for each variety at the same period followed by the same letter are not significantly different at P < 0.05. level. (NAMF) = Control; (AMF) = Mycorrhizal. Cassava varieties: Akoa Essama (AE); Mbong doux (MD); I090590; 96/1414; 92/0326; January 1797; TME/693.

### 3.1 Root yields at harvest

**Fig. 2:**
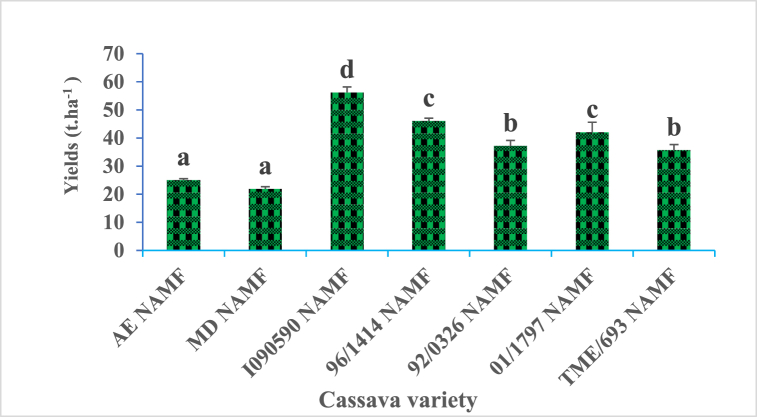
Yield capacity of seven cassava tuber varieties in field condition. (NAMF) = none arbuscular mycorrhizal fungi; Cassava varieties: Akoa Essama (AE); Mbong doux (MD); I090590; 96/1414; 92/0326; January 1797; TME/693.

**Fig. 3:**
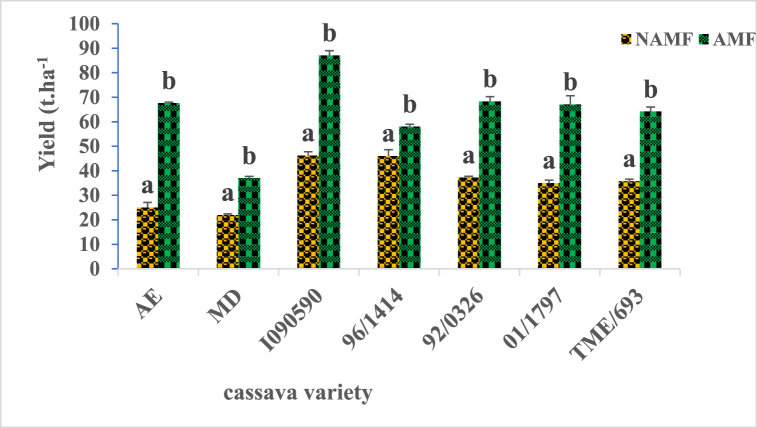
Influence of AM fungi inoculation on tuber yields of cassava varieties in field conditions. *AMF* = *arbuscular mycorrhizal fungi, NAMF* = *none arbuscular mycorrhizal fungi. AE, MD, I090590, 96/1414, 92/0326, 01/1797, and TME/693 are cassava varieties. Bars with the same letter for each variety are not significantly different at P < 0.05*.

Tuber yield varies significantly (P ≤ 0.05) with respect to the cassava variety (Fig. 2). The best yield was observed with variety I090590 and the lowest with variety AE, and MD. Cassava varieties can be classified into four groups according to their yields as follows: I090590; 96/1414, January 1797; 92/0326, TME/693; AE, MD. A significant difference (P ≤ 0.05) of cassava yields with respect to the inoculation status of the plant was recorded for all the varieties of cassava plants (Fig. 3). The ratios of yield increases ranging from 1.24 to 2.7 values respectively for the 96/1414 and AE varieties. The first three cassava varieties that show a better response to inoculation by AM fungi on tuber yield are AE, 01/1797, and I090590 respectively with increase ratios of 2.7, 1.91, and 1.89.

### 3.2 Economic profitability of cassava varieties

**Fig. 4:**
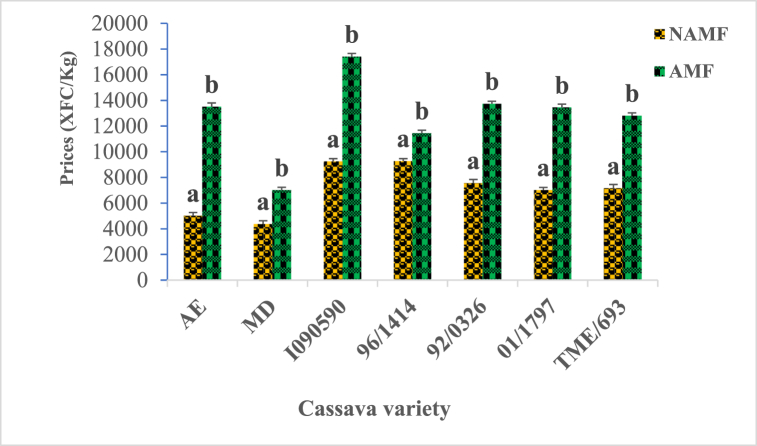
Influence of AM fungi inoculation on the profitability of cassava varieties in field conditions. AMF = arbuscular mycorrhizal fungi, NAMF = none arbuscular mycorrhizal fungi. AE, MD, I090590, 96/1414, 92/0326, 01/1797, and TME/693 are cassava varieties. Bars with the same letter for each variety are not significantly different at P < 0.05.

The conversion of tuber yields of cassava varieties into local currency (XFCA) shows that the gain varies according to the variety used. Variety I090590 is the most profitable and variety AE, and MD are the least profitable. after inoculation with AM fungi, a profound modification of the gain per variety is observed. The initially last variety AE is now among the best in terms of profitability. (Fig. 4).

**Figure: 5:**
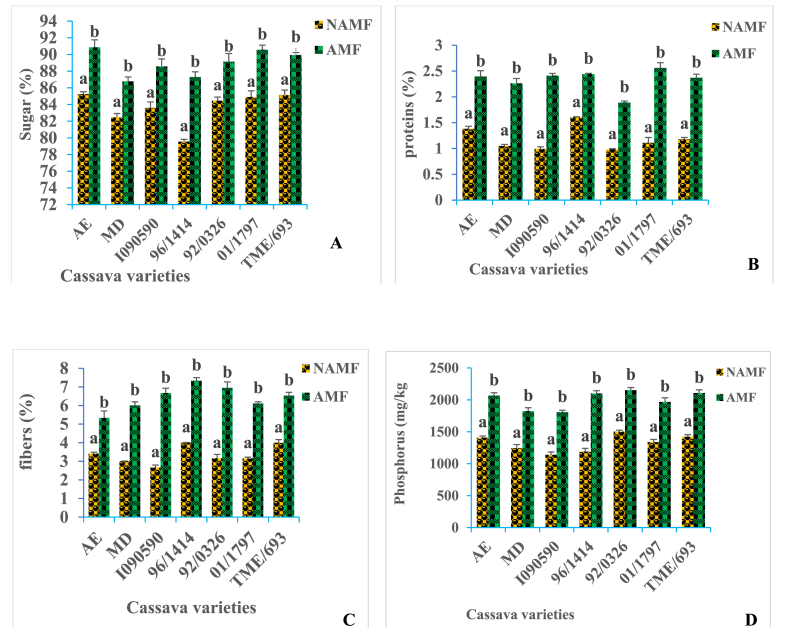
Variation of AM fungi inoculation, and cassava varieties on sugar (A), protein (B), fibers (C), and phosphorus (D) content of tubers in field conditions. *AMF* = *arbuscular mycorrhizal fungi, NAMF* = *none arbuscular mycorrhizal fungi. AE, MD, I090590, 96/1414, 92/0326, 01/1797, and TME/693 are cassava varieties. In graph A,B,C,D, bars with the same letter for each variety are not significantly different at P < 0.05*.

The sugars, proteins, fibers, and phosphorus content of tuber was shown to vary according to the cassava varieties. A significant increase (P < 0.05) of these biomolecules in tubers was observed with all cassava varieties following inoculation by AM fungi (Fig. 5). The best increase was recorded with 96/1414, January 1797 and I090590, I090590, and 96/1414 varieties respectively for sugars, proteins, fibers, and phosphorus.

The cyanide, oxalate, and phytate content was shown to vary in cassava tuber with respect to the varieties. A significant decrease (P ≤ 0.05) with respect to the AM fungi inoculation of the plant was recorded for all the cassava varieties (Table 4). Decrease ratios of cyanide in cassava tuber range from 1.43 to 1.91 values respectively for the 96/1414 and MD varieties.

### 3.3 Principal component analysis of the various parameters and treatments

**Fig. 6:**
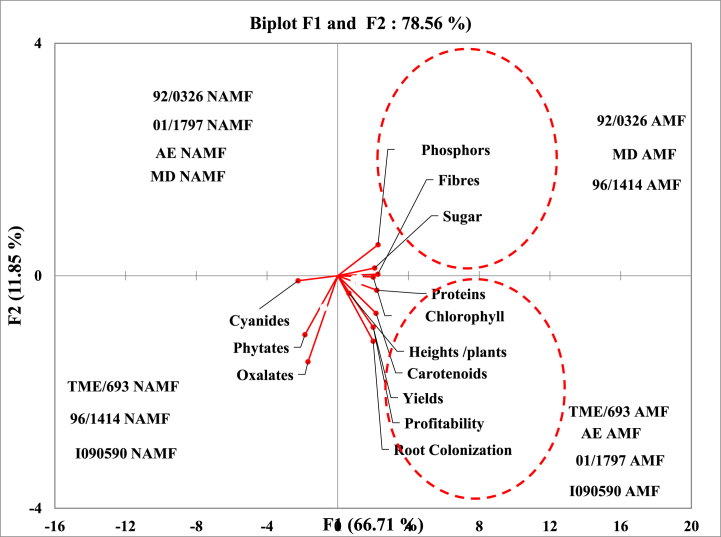
Principal component analysis of the various parameters and treatments.

The Principal Component Analysis of the different cassava varieties with respect to treatment and studied parameters is presented in Fig. 6. The outcome from this graph showed that data are 66.71 %, and 11.85 % consistent with F1, and F2 axis respectively. Globally cassava varieties can be ranged into two main groups according to their inoculation status (AM fungi inoculated or not). With respect to the studied parameters, cassava varieties can be ranged into four groups as follow: group 1 is made up of TME/693, AE, 01/1797, and I090590 varieties all inoculated with AM fungi which showed a high relationship with protein, chlorophyll, carotenoids, as well as yields profitability and root colonization. Pearson correlation (Table 6) point out correlation (P ≤ 0.05) between root colonization and total chlorophyll, carotenoids, yields, and proteins, with respective correlation coefficient (r^2^) of 0.67, 0.86, 0.83, 0.77. The same observation was also noted between yields and proteins, chlorophyll, and carotenoid with respective correlation coefficients (r^2^) of 0.74, 0.61, and 0.84. A correlation was also recorded between chlorophyll and yield, carotenoid, and protein with respective coefficients (r^2^) of 0.61, 0.77, and 0.86.

Group 2 is made up of 92/0326, MD, and 96/1414 varieties all inoculated with AM fungi which showed a high relationship with phosphorus, fiber, and sugar. Pearson positive correlation was recorded (P ≤ 0.05) between phosphorus, fiber, and sugar, with respective coefficients (r^2^) of 0.91, and 0.89. Fiber and sugar are also correlated with a coefficient (r^2^) of 0.73.

Group 3 is made up of TME/693, I090590, and 96/1414 varieties all none inoculated with AM fungi which showed a high relationship with cyanide, phytate, and oxalate. Pearson analysis showed a correlation (P ≤ 0.05) between cyanide and oxalate, and phytate, with respective correlation coefficients (r^2^) of 0.60, 0.74.

Group 4 is made up of 92/0326, MD, and AE, January 1797 varieties all none inoculated with AM fungi which are not linked to any of the analyzed parameters.

### 3.4 Discussion

Structures characteristic of mycorrhizal symbiosis was observed in the roots of all the cassava varieties used in this study with variable colonization percentages, depending on the variety. Following AM fungi inoculation, cassava varieties displayed a significant increase in root colonization percentage (p ≤ 0.05) (Fig. 1), with the best increase ratio of 2.03, recorded for AE varieties. As stipulated in previous studies, cassava belongs to approximately 90 % of terrestrial plant species able to form symbiotic associations with AM fungi [33,34] but many factors can govern the rate of colonization, including environmental factors, plant varieties and more. Such association has a crucial role in plant physiology and nutrition status, management of various stresses bearing its environment, as well as having numerous beneficial uses in agriculture [35]. Characteristic parameters of cassava growth and metabolism were evaluated within the growth, with respect to varieties and AM fungi status. They include chlorophyll content, plant height, carotenoid content, sugar, fiber, phosphorus, and protein content. Chlorophyll content was recorded to vary considerably according to cassava varieties at various periods of growth, with significant increases (p ≤ 0.05) following plants' inoculation with AM fungi (Table 2). The nine-month-old plants were the period at which the best chlorophyll content for all the varieties was recorded. It is established that chlorophyll is a pigment used by plants for the synthesis of carbon molecules within photosynthesis. This observation suggests the implication of genetic factors belonging to each variety, which may have impacted the biosynthesis of chlorophyll in each cassava plant within the growth. Such observation corroborates findings from other studies, indicating that this pigment displays different content concerning genotypes in upland and inland ecology [36,37]. Increases in chlorophyll content following AM fungi inoculation may be one of the numerous benefits gained by cassava plants within the growth and falls in line with other studies raising the implication of varieties on the response to AM fungi inoculation with the increases of chlorophyll content [38,39]. The growth characteristic of the cassava plant greatly increases across varieties with the best performance recorded at 9 months old. The significant positive influence of AM fungi was simultaneously observed for all the cassava varieties with respect to the period of growth (p ≤ 0.05) (Table 3). Carotenoid content shows the same tendency with considerable variation with respect to cassava varieties at various periods of growth, with significant increases (p ≤ 0.05) following plants’ inoculation with AM fungi (Table 4). The nine-month-old plants were the period at which the best carotenoids content for all the varieties was recorded. This observation not only suggests the implication of genotype factors but also the positive effect of the AM fungi symbiosis, which may have boosted the physiology of the plant, leading to the biosynthesis of carotenoid molecules in cassava root within the growth. Studies on tomato plants reveal the increase of carotenoid molecules following AM fungi inoculation [[40], [41], [42], [43]]. The cassava tuber yield is highly variable depending on the variety used in this study, with the best results being recorded for the I090590 variety, an outcome of breeding, and the lowest results with the MD and AE varieties which are wild varieties (Fig. 2, Fig. 3). Such observations suggest the involvement of genotype in the production capacities of cassava varieties and corroborate other studies that record in many trials that yield variations of cassava are a function of factors including genetics, environment, and more [[44], [45], [46]]. This study also shows the significant increase in root yields of each cassava variety following AM fungi inoculation (Fig. 3). This finding suggests a relationship between genotypes and yield variation across cassava varieties, as well as a link with soil-related factors generally improved by AM fungi with a positive influence on plant production. Other studies on cassava shoot biomass and tuber yield recorded high variation across genotypes, strongly linked to soil properties [45,47]. Similarly it was shown that AM fungi strongly impact soil physicochemical properties with consequences on the yields of plants under growth on the involved soil [22,48]. The increase in yield of all the varieties of cassava in this study reflects better profitability regarding the average cost per kilogram on the local market for potential local producers of this commodity (Fig. 4). It is then important for those involved in the production chain of cassava plants to include AM fungi in their production habits. This work shows a significant increase in the content of nutrients such as sugar, protein, phosphorus, and total fiber, in all cassava varieties following inoculation with AM fungi (Fig. 5). Generally, AM fungi are credited with the function of mineral acquisition, especially P, as well as hole plant metabolism improvement, with high evidence on the density of nutrients (sugar, protein), and productivity. Several previous studies report in cassava and other crops that AM inoculation can improve plant biomass, growth, yield, and quality as well as bio transporters of nitrogen phosphorus which guarantee the best concentration of such nutrients in plants [41,49,50]. Cyanides, phytates, and oxalates are biomolecules generally known as antinutrients with the most redoubtable being cyanides. Analysis of their content in this work revealed a large variation with respect to cassava varieties. A significant decrease (p ≤ 0.05; Table 5) was recorded following AM fungi inoculation with all varieties with a remarkable impact on cyanide content. This observation shows additional proof of genotype implication in the synthesis of such molecules in cassava plants mediated by AM fungi inoculation, with an impact on the metabolism leading to such observation. Such finding was recorded in a study on cassava plants (var 92/0326) showed that inoculation of these plants with AM fungi decreases the content of Cyanide as well as phytates, oxalates, and saponins in tubers, which may be their influence on the physiology of the cassava plant during its growth [22,51]. Principal component analysis allows us to group treatments of this study as AM fungi inoculated, and none AM fungi inoculated (Fig. 6). This observation supports the pivotal roles of those microorganisms in this study and suggests their use for the production purpose of cassava. Moreover, analysis of parameters allows us to group the treatments of this study firstly as TME/693, AE, 01/1797, and I090590 all inoculated with AM fungi showing high correlation with physiological and yield characteristic parameters. This suggests that producer mostly use those varieties when they are focusing on the production yield at the end of their trials. Secondly, 92/0326, MD, and 96/1414 were all inoculated with AM fungi correlated with the key mineral nutrient P, sugar, and fiber. This observation suggests that producers should mostly focus on those varieties when they intend to high phosphorus, sugar, or fiber cassava tuber yields. The third group is TME/693, 96/1414, and I090590 not AM fungi inoculated which showed a high correlation with antinutrients including cyanides. This observation suggests a biotechnological way to drop down the amount of those antinutrients in cassava tubers at harvest and should be considered by the producer.

**Table 5:** Cyanides, oxalates, and phytates tubers content of cassava varieties as response to AM fungi inoculation in field conditions.

Antinutrients (ppm)			
Varieties	Cyanides	Oxalates	Phytates
AE (AMF)	195.73 ± 0.72^a^	0.65 ± 0.02^a^	0.21 ± 0.05^a^
AE (NAMF)	335.20 ± 0.40^b^	0,86 ± 0.01^b^	0.29 ± 0.01^b^
MD (AMF)	198.27 ± 0.46^a^	0,63 ± 0,05^a^	0.27 ± 0.01^a^
MD (NAMF)	377.87 ± 0.61^b^	1.19 ± 0.02^b^	0.35 ± 0.05^b^
I090590 (AMF)	193.47 ± 0.83^a^	0.96 ± 0.01^a^	0.240 ± 0.01^a^
I090590 (NAMF)	345.13 ± 0.42^b^	1.77 ± 0.01^b^	0.37 ± 0.02^b^
96/1414 (AMF)	173.60 ± 0.80^a^	0.66 ± 0.05^a^	0.26 ± 0.02^a^
96/1414 (NAMF)	247.86 ± 0.46^b^	1.55 ± 0.01^b^	0.337 ± 0.03
92/0326 (AMF)	174 ± 0.26^a^	0.31 ± 0.01^a^	0.18 ± 0.05^a^
92/0326 (NAMF)	308 ± 0.92^b^	0.61 ± 0.01^b^	0.23 ± 0.01^b^
January 1797 (AMF)	163.83 ± 0.67^a^	0.56 ± 0.02^a^	0.18 ± 0.03^a^
January 1797 (NAMF)	270.8 ± 0.80^b^	0.873 ± 0.01^b^	0.24 ± 0.02^c^
TME/693 (AMF)	152 ± 0.8^a^	0.413 ± 0.05^a^	0.21 ± 0.01^b^
TME/693 (NAMF)	246 ± 0.6^b^	1.420 ± 0.01^b^	0.27 ± 0.03^d^

Data in Column for each variety at the same period followed by the same letter are not significantly different at P < 0.05. level. (NAMF) = Control; (AMF) = Mycorrhizal. Cassava varieties: Akoa Essama (AE); Mbong doux (MD); I090590; 96/1414; 92/0326; January 1797; TME/693.

**Table 6:** Pearson correlation matrix between the studied parameters for the different varieties.

Variables	Colonization	Chlorophyll	Carotenoid	Yield	Profitability	Height	Sugar	Fiber	Phosphorus	Protein	Cyanide	Oxalate	Phytate
Colonization	1	0,6733	0,8652	0,8339	0,8339	0,4323	0,7296	0,7818	0,7045	0,7790	−0,8004	−0,2687	−0,4560
Chlorophyll	0,6733	1	0,7707	0,6135	0,6135	0,1879	0,6506	0,8620	0,8151	0,8665	−0,7803	−0,5247	−0,4820
Carotenoid	0,8652	0,7707	1	0,8465	0,8465	0,2546	0,7390	0,8668	0,7892	0,8798	−0,8005	−0,5036	−0,5556
Yield	0,8339	0,6135	0,8465	1	1,0000	−0,0155	0,6904	0,7537	0,6962	0,7437	−0,7569	−0,3627	−0,5803
Profitability	0,8339	0,6135	0,8465	1,0000	1	−0,0155	0,6904	0,7537	0,6962	0,7437	−0,7569	−0,3627	−0,5803
Height	0,4323	0,1879	0,2546	−0,0155	−0,0155	1	0,2719	0,2749	0,2178	0,3151	−0,3472	−0,1054	−0,0868
Sugar	0,7296	0,6506	0,7390	0,6904	0,6904	0,2719	1	0,7344	0,8923	0,7573	−0,7356	−0,7452	−0,8217
Fiber	0,7818	0,8620	0,8668	0,7537	0,7537	0,2749	0,7344	1	0,9132	0,9101	−0,9096	−0,6469	−0,6393
Phosphorus	0,7045	0,8151	0,7892	0,6962	0,6962	0,2178	0,8923	0,9132	1	0,8534	−0,8633	−0,8290	−0,8005
Protein	0,7790	0,8665	0,8798	0,7437	0,7437	0,3151	0,7573	0,9101	0,8534	1	−0,8765	−0,5769	−0,5733
Cyanide	−0,8004	−0,7803	−0,8005	−0,7569	−0,7569	−0,3472	−0,735	−0,909	−0,8633	−0,8765	1	0,6040	0,7418
Oxalate	−0,2687	−0,5247	−0,5036	−0,3627	−0,3627	−0,1054	−0,745	−0,646	−0,8290	−0,5769	0,6040	1	0,8542
Phytate	−0,4560	−0,4820	−0,5556	−0,5803	−0,5803	−0,0868	−0,821	−0,639	−0,8005	−0,5733	0,7418	0,8542	1

## 4 Conclusion

This study clearly points out the variation of the rate of growth, nutritional, and antinutritional amount as well as the production yield and profitability of cassava tubers with respect to the varieties. AM fungi inoculation greatly improving cassava plant growth and increases nutritional properties, yield, and gain of all the varieties with the best being I090590. In contrast, AM fungi inoculation significantly decreases the amount of antinutrients in the tuber of the studied varieties of cassava. Bio-fertilization with AM fungi must be an important way to ensure safe, exponential production, and high economic profitability of foodstuffs. Moreover, plant inoculation with AM Fungi should be one of the key tools driving plant varietal selection.

## CRediT authorship contribution statement

**Mbassi Manga Gilbert Ghislain:** Methodology, Investigation, Formal analysis, Conceptualization. **Djontzo Toche Emmilienne:** Methodology, Investigation, Formal analysis. **Essono Damien Mari:** Methodology, Conceptualization. **Adamou Souleymanou:** Methodology, Investigation, Formal analysis, Conceptualization, Methodology, Formal analysis, Data curation. **Fokom Raymond:** Methodology, Investigation, Formal analysis, Data curation, Conceptualization. **Nouhou Abassi:** Methodology, Formal analysis, Data curation. **Noah Guy:** Methodology, Formal analysis, Data curation. **Sonkeng Aurelie:** Methodology, Investigation, Formal analysis, Data curation, Conceptualization. **Nwaga Dieudonné:** Methodology, Investigation, Formal analysis, Data curation. **Fokou Elie:** Methodology, Investigation, Formal analysis, Conceptualization.

## Declaration of competing interest

The authors declare that they have no known competing financial interests or personal relationships that could have appeared to influence the work reported in this paper.
